# Prevention of Chronic Hepatitis B after 3 Decades of Escalating Vaccination Policy, China

**DOI:** 10.3201/eid2305.161477

**Published:** 2017-05

**Authors:** Fuqiang Cui, Lipin Shen, Li Li, Huaqing Wang, Fuzhen Wang, Shengli Bi, Jianhua Liu, Guomin Zhang, Feng Wang, Hui Zheng, Xiaojin Sun, Ning Miao, Zundong Yin, Zijian Feng, Xiaofeng Liang, Yu Wang

**Affiliations:** Peking University Health Science Center, Beijing, China (F. Cui);; Chinese Center for Disease Control and Prevention, Beijing (L. Shen, L. Li, H. Wang, F. Wang, S. Bi, G. Zhang, F. Wang, H. Zheng, X. Sun, N. Miao, Z. Yin, Z. Feng, X. Liang, Y. Wang);; Guangzhou Center for Disease Control and Prevention, Guangzhou, China (J. Liu)

**Keywords:** hepatitis B, vaccine, vaccination, immunization, China, viruses

## Abstract

China’s hepatitis B virus (HBV) prevention policy has been evaluated through nationally representative serologic surveys conducted in 1992 and 2006. We report results of a 2014 serologic survey and reanalysis of the 1992 and 2006 surveys in the context of program policy. The 2014 survey used a 2-stage sample strategy in which townships were selected from 160 longstanding, nationally representative, county-level disease surveillance points, and persons 1–29 years of age were invited to participate. The 2014 sample size was 31,713; the response rate was 83.3%. Compared with the 1992 pre–recombinant vaccine survey, HBV surface antigen prevalence declined 46% by 2006 and by 52% by 2014. Among children <5 years of age, the decline was 97%. China’s HBV prevention program, targeted toward interrupting perinatal transmission, has been highly successful and increasingly effective. However, this progress must be sustained for decades to come, and elimination of HBV transmission will require augmented strategies.

Hepatitis B virus (HBV) causes ≈240 million chronic infections and ≈780,000 deaths from cirrhosis and liver cancer annually (*1*). Recognizing the large global disease burden, the United Nations Sustainable Development Goals for 2030 include combating hepatitis. HBV has been highly endemic in China, where serosurveys in 1979 and 1992 indicated a 10% prevalence of HBV surface antigen (HBsAg) (*2*,*3*). High rates of chronic HBV infection among infants indicated that the infection occurred in early childhood (*4*–*6*). Historical HBV transmission built a reservoir of ≈90 million chronically infected persons in China (*7*,*8*), accounting for 30% of the global burden of chronic HBV infection (*9*).

The government of China adopted increasingly comprehensive strategies to prevent HBV transmission, including immunization, promotion of safe injection practices, blood donation screening, and surveillance (*10*). Implementation of China’s immunization strategy began in 1985 with licensure of plasma-derived hepatitis B vaccine (HepB). A recombinant vaccine was licensed in 1992 and managed nationally. The strategy was designed to interrupt perinatal HBV transmission and provide newborns lifelong protection from HBV with a birth dose of HepB followed by 2 additional doses during infancy. Before 2002, HepB was managed as a type 2 vaccine for which parents or adult vaccinees had to pay out-of-pocket. In 2002, China integrated HepB into its Expanded Program on Immunization (EPI), making the vaccine available at no cost to children through 14 years of age (*11*,*12*). During 2009–2011, China conducted a HepB catch-up campaign for children <15 years of age who were born during 1994–2001; this campaign vaccinated ≈68 million children with HepB. In 2011, China launched a program integrating prevention of mother-to-child transmission of HIV, syphilis, and HBV in 1,156 counties (representing 44% of pregnant women in China) and then expanded the program nationwide in 2015, covering all pregnancies (*13*–*15*).

The effectiveness of China’s HBV prevention measures is evaluated by using national serologic surveys; the fourth such survey was conducted in November 2014. We report results of this survey in the context of China’s HBV prevention and control measures, along with reanalysis of the 1992 and 2006 surveys (*3*,*7*).

## Methods

### Survey Conduct

Target populations for all 3 surveys were local residents residing in national disease surveillance points (DSPs) for >6 months. DSPs were selected by the Chinese Academy of Preventive Medicine (now the Chinese Center for Disease Control and Prevention [China CDC]) to be representative of the population of China. Population demographic and socioeconomic conditions and morbidity and mortality continue to be representative (*16*–*18*).

The surveys’ targeted age ranges were 1–59 years in 1992 and 2006 and 1–29 years in 2014. The 2014 serosurvey used age groups of 1–4 years, 5–14 years, and 15–29 years; we reanalyzed the 1992 and 2006 surveys by using these groupings.

The 1992 serosurvey used a multistage cluster sampling strategy. Three villages were identified at random from each of 145 DSPs; families were randomly selected from each village from lists of residents; and all age-appropriate family members were selected (*3*,*19*). The total sample size was 67,017.

The 2006 serosurvey used a 3-stage cluster sampling strategy to identify 369 townships at random for the first stage and 369 villages at random for the second stage. Finally, 81,775 persons, stratified into age groups of 1–4 years, 5–14 years, and 15–59 years, were selected at random from a list of village residents (*7*,*20*). Children 1–14 years of age were oversampled to increase the precision of estimates for young children.

The 2014 serosurvey used a 2-stage cluster random sample from the same 160 DSPs that were used in the 2006 serosurvey. First, we allocated the same proportion of the sample to each region (eastern, middle, and western) and location type (urban and rural); second, in each region, we allocated samples to each DSP by using a probability-proportional-to-size method; third, in each DSP, we randomly selected 3 villages or communities, and from these, samples in grouping of 1–4 years, 5–14 years, and 15–29 years from each village or community were selected based on simple random sample. In all, 324 villages/communities were selected from the 38,527 villages/communities in the DSPs, with a sampling probability proportional to their size. Then persons were selected by simple random sample from local government lists of residents of sampled villages/communities into the age-group strata. The sample size calculation was based on expected HBsAg prevalence extrapolated from the 2006 survey by age group (0.7% for 1–4 years, 1.5% for 5–14 years, and 5.0% for 15–29 years) and was powered to detect differences of +50% the expected point prevalence. The final target sample size was 31,024. Lists of eligible persons were sampled systematically until the target sample size was reached.

The field investigation methods were identical in the 3 serosurveys (*3*,*7*). The interviews were carried out by trained professionals through house-to-house visits in the order of the sample listings. Communities had been notified in advance of the survey. Working persons and school children were interviewed during weekends or after school hours. For persons who were not at home, the interview staff made up to 3 additional home visits within 1 week. If, after 3 unsuccessful visits, the person could not be found, he or she was considered missing. Face-to-face interviews with the respondent or the respondent’s parent were completed by trained staff by using standard questionnaires to obtain basic information, including sex, birthdate, ethnicity, birthplace, and HepB vaccination history of the children <15 years of age (validated by parent-held certificate or village vaccination record).

### Laboratory Technique

All specimens were tested in the National Hepatitis Laboratory of the Institute for Viral Disease Control and Prevention, China CDC. For the 2006 (*7*,*20*) and the 2014 serosurveys, ELISA reagents were used to detect levels of HBsAg, anti-HBV surface antigens (anti-HBs), and anti-HBV core antigens (anti-HBc). HBsAg >2.1 IU was considered positive for consistency across serosurveys. Specimens yielding inconsistent or indeterminate results were retested by using microparticle enzyme immunoassay reagents (Abbott Laboratories, Chicago, IL, USA). HBsAg-positive specimens were tested for HBV e antigen and anti–HBV e antigen also by using Abbott microparticle enzyme immunoassay reagents. For the 1992 serosurvey, HBsAg, anti-HBs, and anti-HBc were tested by solid-phase radioimmunoassay (SPRIA) (*3*,*19*).

### Statistical Analysis

In the 2006 and 2014 surveys, data were double-entered into EpiData version 3.02 (EpiData Association, Odense, Denmark) and verified for consistency and then were analyzed by using SAS version 9.4 (SAS Institute, Inc., Cary, NC, USA). Statistical methods of the 2014 serosurvey were identical to those of the 2006 serosurvey. To ensure representativeness of poststratification adjustments, sample weighting components were village selection probability and age-specific and person-selection probabilities within the village. The weight per person *i* was *w_ji_ = w_j_× w_i,j_× w_adj_*, where *w_j_* was the reciprocal of the probability of including village *j*, *w_i,j_* was the reciprocal of the conditional inclusion probability of person *i* from village *j*, and *w_adj_* was an adjustment factor for person *i* so the sum of weights equaled China’s population. We used the SAS procedure surveyfreq to calculate point estimates and 95% CIs of serologic markers by using weighting adjustments; Taylor series linearization was used for variance estimations. The 1992 survey had no design weighting, so we determined unweighted point prevalence and 95% CIs.

The HBsAg, anti-HBs, and anti-HBc prevalence of persons 1–29 years of age covered in the 1992 and 2006 serosurveys were reanalyzed to be consistent with the format of the 2014 serosurvey by 3 age groups (1–4 years, 5–14 years, and 15–19 years), as well as sex, ethnicity, location type (urban or rural), region, and year of birth.

### Vaccination Coverage and HBsAg Prevalence by Birth Year

HepB vaccination history for children <15 years of age was coded as vaccinated (i.e., birth dose plus 2 more doses or incomplete series), unvaccinated, or unknown, based on children’s immunization certificates. Coverage levels of children born during 1985–1991, 1992–2005, and 2006–2013 were determined from the 1992, 2006, and 2014 surveys, respectively. Weighted HBsAg prevalences for the 1962–1991, 1976–2005, and 1985–2013 birth cohorts were determined by using the 1992, 2006, and 2014 surveys for each birth cohort included in the respective surveys.

### Analyses of Cases Averted

We estimated the number of chronic HBV infections prevented in the 1992–2013 birth cohorts by using Goldstein’s model, which was used in the 2006 survey to estimate baseline disease prevalence and cases prevented (*21*). This model provides estimates of total numbers of cases and deaths caused by acute HBV infection and numbers of cases and sequelae from chronic HBV infection, including cirrhosis and primary hepatocellular carcinoma that would develop during the lifetime of a birth cohort. The key inputs to the model are baseline HBsAg seroprevalence in the entire population and among women of childbearing age and HepB coverage. Figures on the effect of vaccination by birth cohort were summed to estimate the overall effect by using birth cohort sizes of 16.97 million persons per year. We assumed a baseline HBsAg prevalence of 8.58% among women of childbearing age, uniformly distributed, with 30% also being HBeAg positive. Among 5-year-old children, 32% were assumed to become chronically HBV-infected (anti-HBc positive) by 5 years of age and 55% to be chronically infected by 30 years of age; these percentages represented force of infection without vaccination (*6*,*20*).

### Quality Control

China CDC convened expert groups to guide design, fieldwork, laboratory testing, and analyses for the 3 surveys. Pilots were conducted before each survey. County CDC staff administered questionnaires and collected and managed blood specimens.

### Ethical Reviews

The 1992 survey was approved by Chinese Academy of Preventive Medicine’s Ethical Review Committee; the 2006 and 2014 surveys were approved by China CDC’s Ethical Review Committee. In 2006 and 2014, participants were informed of the study purpose and their right to keep information confidential. Consent was obtained before interview and blood drawing.

## Results

### Response Rate

**Figure 1:**
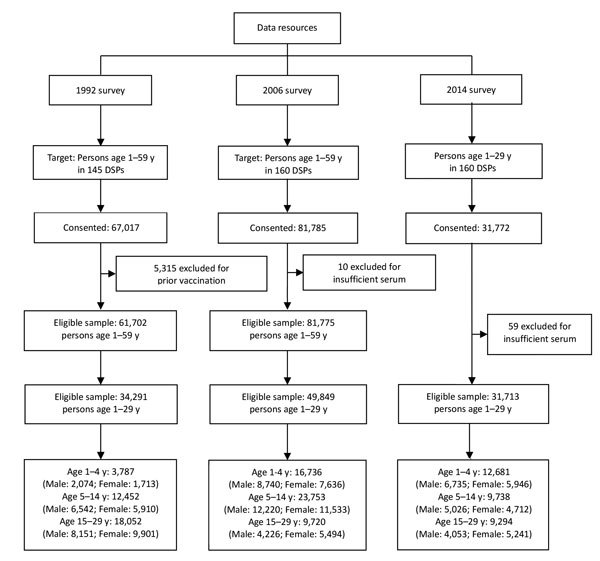
Study eligibility and select characteristics of persons participating in 1992, 2006, and 2014 national serosurveys for hepatitis B virus, China. DSPs, disease surveillance points.

In the 2014 survey, investigators visited selected houses up to 3 times and invited 38,142 persons to participate. Of those invited, 31,772 gave consent, yielding an 83.3% response rate. Among those consenting, 59 (0.2%) were excluded because of insufficient serum for laboratory analysis. The final sample was 31,713 persons (Figure 1). Demographic characteristics of the subjects in the 3 surveys were similar except that the percentage of the sample residing in urban areas increased from 25.7% to 49.6% during 1992–2006, and 49.8% in 2014, reflecting China’s urbanization (Technical Appendix Table 1). Among respondents <15 years of age in the 1992, 2006, and 2014 surveys, 96.5% (21,638 of 22,419), 81.6% (32,732 of 40,129), and 18.0% (2,923 of 16,239) had vaccination records, respectively.

### HBV Serologic Markers

HBsAg prevalence among persons 1–4 years, 5–14 years, and 15–29 years of age in 2014 was 0.3%, 0.9%, and 4.4%, respectively. We compared HBsAg, anti-HBsAg, and anti-HBc results from the 3 surveys (Technical Appendix Table 2). HBsAg prevalence among 1–29-year-olds declined from 10.1% to 2.6% during 1992–2014. Declines were observed in all age, sex, ethnicity, location type (urban/rural), and regional groups. Among children <15 years of age, HBsAg prevalence declined from 10.5% to 0.8%. Prevalence of anti-HBs among 1–29-year-olds increased from 25.4% to 57.8% during 1992–2014. Prevalence of anti-HBc declined from 45.8% to 13.0% during 1992–2014, declining in all subpopulations.

**Figure 2:**
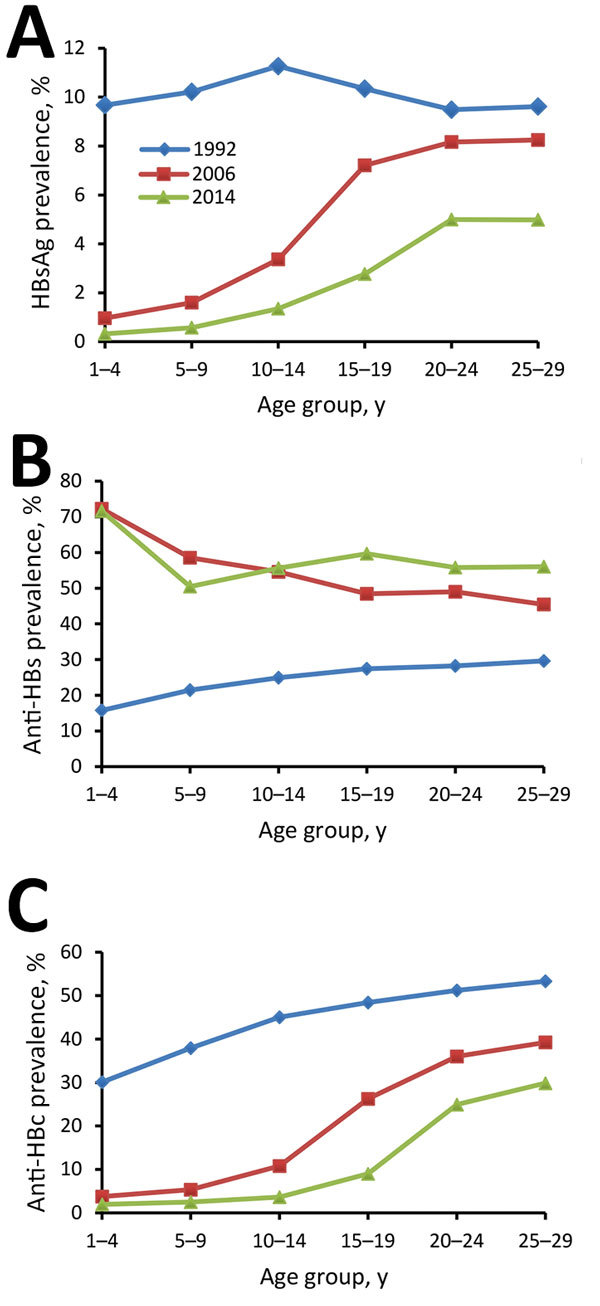
Longitudinal changes in prevalence of HBsAg (A), anti-HBs (B), and anti-HBc (C) among persons participating in 1992, 2006, and 2014 national serosurveys for hepatitis B virus, by age group, China. HBsAg, hepatitis B virus surface antigen; anti-HBs, antibody to hepatitis B virus surface antigen; anti-HBc, antibody to hepatitis B virus core antigen.

In 1992, HBsAg prevalence was 10% across all age groups (Figure 2), consistent with HBsAg prevalence in 1979 (*22*). In 2006, HBsAg prevalence was high among 20–29-year-olds (8.3%) and low among 1–4-year-olds (1.0%). Similar trends in HBsAg prevalence were observed seen in the 2014 survey.

**Figure 3:**
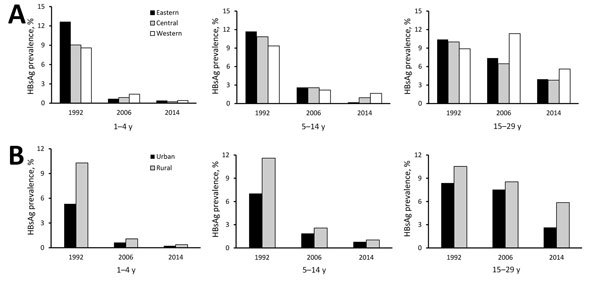
Prevalence of HBsAg by region, age group, and survey year (A) and by location type (urban or rural), age group, and survey year (B) among persons participating in 1992, 2006, and 2014 national serosurveys for hepatitis B virus, China. HBsAg, hepatitis B virus surface antigen.

**Figure 4:**
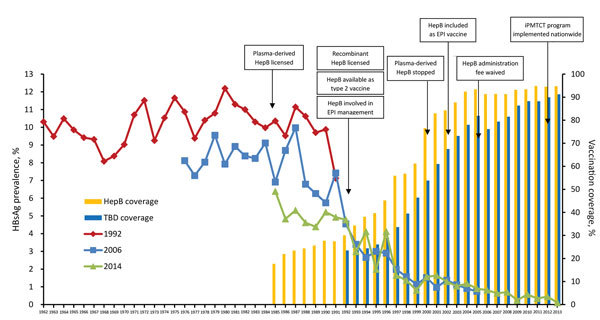
Prevalence of HBsAg and 3-dose HepB coverage for each birth cohort and major vaccination program milestones for hepatitis B virus, China, 1962–2014. HBsAg prevalence is shown in 3 curves, 1 for each national serologic survey (1992, 2006, and 2014). HepB coverage is shown in bars. Type 2 vaccines are private sector vaccines that are not included in the free national EPI system but must be paid for out-of-pocket. HepB coverage was defined as the percentage of children <15 years of age who received 3 doses of HepB before reaching 12 months of age. Coverage levels of children born during 1985–1991, 1992–2005, and 2006–2013 were determined from the 1992, 2006, and 2014 surveys, respectively. TBD coverage was defined as the percentage of newborn infants who received a dose of HepB within 24 hours of birth. The iPMTCT program provides free HBsAg screening of pregnant women and free hepatitis B immunoglobulin for hepatitis B virus–exposed infants. EPI, Expanded Program on Immunization; HBsAg, hepatitis B virus surface antigen; HepB, hepatitis B vaccine; iPMTCT, integrated Prevention of Mother-to-Child Transmission, TBD, timely birth dose.

The relative decline in HBsAg prevalence was uneven by region and age group (Figure 3, panel A). Among 1–4-year-olds, eastern, central, and western region prevalences decreased by >95%, but among 15–29-year-olds, declines were 62.0%, 62.1%, and 37.0%, respectively. The decline in HBsAg prevalence was >95% among 1–4 year-olds regardless of rural/urban status (Figure 3, panel B), but among 15–29-year-olds, the decline was greater among urban residents than rural residents (68.4% and 44.3%). HBsAg prevalence by birth cohort and HepB coverage, when mapped against the timeline of important immunization program events, were noticeably affected as incremental interventions were added (Figure 4). 

### Cases Averted

During 2010–2014, China prevented an additional estimated 4 million chronic HBV infections on top of the 24 million chronic infections prevented during 1992–2009 (*21*). In total, 28 million chronic HBV infections were averted, and 5 million deaths from HBV infection complications were prevented

## Discussion

Compared with the prevaccine era, chronic HBV infection in China has been reduced by 90% (from 10.5% to 0.8%) among children <15 years of age and by 97% (from 9.9% to 0.3%) among children <5 years of age. Disparities by region and urban/rural status that existed among young children in 1992 and 2006 were largely eliminated by 2014. Lower HBsAg prevalence among young children in 2014 (1.0%) compared with 2006 (0.3%) shows increasing effectiveness of the program.

HBsAg prevalence among 1–29-year-olds declined 46% during 1992–2006 (from 10.1% to 5.5%) and 52% during 2006–2014 (from 5.5% to 2.6%). As a result of China’s program, an estimated 120 million HBV infections and 28 million chronic infections were averted.

Without postexposure prophylaxis provided by the HepB birth dose, 30% of infants born to HBsAg-positive mothers will become infected, and 90% of the infections will become chronic (*1*). Because administering a birth dose is challenging when childbirth happens in the home, a key element of China’s success was promoting facility-based childbirth. Implementation of the timely birth dose was accelerated by the GAVI hepatitis B project, which promoted the birth-dose policy in rural and western areas of China (*12*). Additional strategies, such as promoting safe injection practices and screening donated blood, have also been important for chronic HBV prevention. In 2000, China passed a regulation banning the reuse of medical devices labeled for single use. In 2005, the Chinese Medical Association published clinical guidelines for injections and other skin-piercing procedures. In 2007, autodisable syringes became available for vaccine injections, and by 2010, reusable injection equipment was eliminated in China and disposable and autodisable syringes became universally used (*23*). Since 1988, donated blood has been screened for HBV serologic markers, and since 2015, HBsAg-negative donated blood has been tested for HBV DNA (*24*). Although HBV infection caused by unsafe injections and blood transfusion has been reduced, modeling shows that the newborn and infant vaccination strategy has been independently responsible for preventing ≈95% of chronic HBV infections in China (*25*).

The government of China regards health equity as important for social justice and fairness (*26*). In 2000, the ministries of health and finance and the State Council implemented a program to reduce maternal mortality rates and eliminate maternal/neonatal tetanus. The government established insurance plans to ensure access to healthcare and birth facilities, especially in impoverished, remote, or ethnic minority areas. The in-hospital delivery rate increased from 44% in 1985 to 99% in 2013 (*27*). By using the principle “whoever delivers the baby vaccinates the baby,” virtually all infants born in birthing facilities receive a birth dose of HepB (*10*).

Timely vaccination is used as an evaluation measure of public health effectiveness (*5*,*28*–*31*). High 3-dose vaccination coverage has been maintained continuously from 2009 through 2015, having increased from 70% in 2002 to >95% in 2009 and afterward. HepB birth-dose coverage increased from 22% in 1992 to 71% in 2002 and 94% in 2013 (*32*). The national program integrating prevention of mother-to-child transmission of HIV, syphilis, and HBV has been providing HBsAg screening for pregnant women and hepatitis B immunoglobulin for all infants born to HBsAg-positive women since 2012 (*3*,*13*,*33*).

Vaccines used in the program have been evaluated periodically. When HepB became government-supported in 2002, the dose provided was 5 μg/0.5 mL, which was known to prevent infection in 85%–90% of children born to HBsAg-positive women (*34*–*37*). To improve effectiveness, the dose was increased to 10 μg/0.5 mL in 2011. China’s model supports the United Nations Sustainable Development Goals and the World Health Organization’s new Global Hepatitis Framework by greatly reducing HBV transmission with strategies that integrate HBV prevention into the healthcare sector.

Strengths of this study include a sound sampling strategy with comparable methods across 3 surveys separated in time, identical laboratory procedures in the 2006 and 2014 surveys, and use of sufficiently large sample sizes to support precise estimates. Our study provides previously unpublished coverage levels of plasma-derived HepB before licensure of the recombinant vaccine.

Weaknesses of the study include the use of different laboratory methods for the 1992 survey than those used for the 2006 and 2014 surveys, different nonresponse rates of the 2006 and 2014 surveys, underestimation of immunity indicated by anti-HB levels (because when antibody levels wane, memory B cell mediated anamnestic response to HBV exposure can maintain protection from infection), undersampling of the migrant population (because only those residing >6 months in a given survey area were included), and the fact that HepB coverage levels among teens and adults are not measured in China. The 1992 survey used SPRIA for HBV infection serologic markers, and the 2006 and 2014 surveys used ELISA. According to previous studies (*38*,*39*), SPRIA is less specific than ELISA for detecting HBsAg; this difference could have led to overestimation of the relative decrease in HBsAg prevalence because ELISA testing was used in 2006 and 2014. However, we believe that the effect on our results is modest, especially for the current estimates of HBsAg prevalence, because the ELISA tests used are considered acceptable at international standards. We used the same HBsAg cutoff values for ELISA testing in the 2006 and 2014 surveys for the sake of consistency; however, higher cutoff values are used more frequently now.

HBsAg prevalence among several birth cohorts was measured by >1 survey (Figure 4). Of interest is that results are more consistent for younger, double-measured birth cohorts than for older, double- and triple-measured cohorts. These differences might be attributable to several reasons. Persons in the double-measured birth cohorts were 22 years older in the 2014 survey than the 1992 survey and were 8 years older in the 2014 survey than the 2006 survey. HBsAg prevalence has a small, natural decline with age (*40*). This natural decline will increase the HBsAg prevalence differences from the 1992 survey, as will the age-based accumulation of deaths caused by complications of chronic HBV. Finally, lower specificity of the 1992 survey can lead to an upward bias of the differences from the 1992 survey.

Our study has 3 main programmatic implications. First, the annual need for perinatal postexposure prophylaxis remains substantial. The prevalence of HBsAg in women of childbearing age and the size of the birth cohort in China (16.97 million), implies that 750,000 to 1 million infants are born to HBsAg-positive women annually (*13*).

Second, prevention measures must continue for decades. The age group with the highest HBsAg prevalence corresponds to the age groups with the highest fertility rate in China (69.5/1,000 for those 20–24 years and 94.0/1,000 for those age 24–29 years of age) (*27*). Even when children of today become adults, nearly 200,000 infants will be born to HBsAg carriers each year and will need postexposure prophylaxis to prevent HBV infection.

Third, many newborns still become chronically infected. Although China has reduced perinatal transmission by 97%, an HBsAg prevalence of 0.3% in a birth cohort of 16 million implies that 50,000 perinatal infections still occur annually. Additional strategies will be needed to eliminate vertical transmission.

We believe that the success HBV prevention should be communicated to stakeholders to help sustain confidence in the immunization effort. Confidence in vaccines can be fragile, as was made evident by a temporary loss of confidence in HepB in 2013 and 2014 (*41*), and showing the strongly positive impact of vaccination may help maintain or restore confidence (*42*).

Our results raise several questions. Can the current strategy eliminate perinatal transmission? HepB is not 100% effective, and additional strategies may need to be used. Antiviral prophylaxis during the third trimester for HBsAg-positive pregnant women with high HBV DNA is being shown to decrease perinatal transmission of HBV (*43*,*44*) and may need to become a standard of care in the future.

Should postvaccination serologic testing (PVST) become a recommended standard in China? PVST can help confirm whether an HBV-exposed infant is protected, is susceptible and needs to be revaccinated, or is infected and needs referral for follow-up care. The cost-effectiveness, feasibility, and acceptability of PVST for HBV-exposed infants in China should be evaluated.

Can adults at risk of HBV infection be vaccinated? Identifying cost-effective means to protect at-risk adults from HBV has potential to avert infections (*45*).

Finally, treating the estimated 90 million persons with chronic HBV infection is critically important (*9*,*46*). Prevention works, but not always perfectly, and many adults were born before prevention of HBV was possible.

Technical Appendix Number and percentage of persons participating in national serosurveys and HBsAg, anti-HBs, and anti-HBc prevalence, by survey year and selected characteristics, China, 1992, 2006, and 2014.
