# Supplementary material for: Prevention of Chronic Hepatitis B after 3 Decades of Escalating Vaccination Policy, China
Source: Emerg Infect Dis. 2017 May;23(5):765–72. doi: 10.3201/eid2305.161477 (PMC5403029; doi:10.3201/eid2305.161477)
Supplement: Technical Appendix — Number and percentage of persons participating in national serosurveys and HBsAg, anti-HBs, and anti-HBc prevalence, by survey year and selected characteristics, China, 1992, 2006, and 2014. [file 16-1477-Techapp-s1.pdf]

Article DOI: http://dx.doi.org/10.3201/eid2304.161477

Prevention of Chronic Hepatitis B after 3 Decades of Escalating Vaccination Policy, China

Technical Appendix

Technical Appendix Table 1. Number and percentage of persons participating in the 1992, 2006, and 2014 national serosurveys in China, by selected characteristics

	No. (%)		
Characteristic	1992	2006	2014
Age group, y			
1–4	3,787 (11.0)	16,376 (32.9)	12,681 (40.0)
5–14	12,452 (36.3)	23,753 (47.6)	9,738 (30.7)
15–29	18,052 (52.7)	9,720 (19.5)	9,294 (29.3)
Sex			
Male	16,767 (48.9)	25,186 (50.5)	15,814 (49.9)
Female	17,524 (51.1)	24,663 (49.5)	15,899 (50.1)
Ethnicity			
Han	30,324 (88.4)	42,759 (85.8)	26,781 (84.4)
Mongolian	385 (1.1)	389 (0.8)	373 (1.2)
Tibetan	752 (2.2)	1,567 (3.1)	1,427 (4.5)
Uyghur	406 (1.2)	868 (1.7)	557 (1.8)
Zhuang	486 (1.4)	312 (0.6)	283 (0.9)
Muslim	453 (1.3)	750 (1.5)	484 (1.5)
Other	1,485 (4.4)	3,204 (6.5)	1,808 (5.7)
Location type			
Urban	8,813 (25.7)	24,802 (49.8)	15,739 (49.6)
Rural	25,478 (74.3)	25,047 (50.2)	15,974 (50.4)
Region			
Eastern	10,541 (30.7)	16,546 (33.2)	10,424 (32.9)
Central	14,067 (41.0)	16,555 (33.2)	10,362 (32.7)
Western	9,683 (28.3)	16,748 (33.6)	10,927 (34.4)
Total	34,291 (100.0)	49,849 (100.0)	31,713 (100.0)

Technical Appendix Table 2. HBsAg, anti-HBs, and anti-HBc prevalence among persons participating in the 1992, 2006, and 2014 national serosurveys in China, by selected characteristics*

	HBsAg prevalence (95% CI)			Anti-HBs prevalence (95% CI)			Anti-HBc prevalence (95% CI)		
Characteristic	1992	2006	2014	1992	2006	2014	1992	2006	2014
Age group, y									
1–4	9.9 (9.0–10.9)	1.0 (0.8–1.2)	0.3 (0.2–0.5)	16.0 (14.8–17.2)	71.2 (69.8–72.7)	71.6 (70.1–73.2)	31.5 (30.0–33.0)	4.1 (3.5–4.8)	2.0 (1.4–2.7)
5–14	10.6 (10.1–11.2)	2.42 (2.1–2.9)	0.9 (0.7–1.3)	23.5 (22.8–24.2)	56.9 (54.9–58.6)	52.9 (50.7–55.0)	41.9 (41.1–42.8)	8.4 (7.6–9.2)	3.0 (2.5–3.7)
15–29	9.8 (9.4–10.3)	8.4 (7.0–10.1)	4.4 (3.8–5.1)	28.7 (28.1–29.4)	47.5 (44.7–50.3)	56.9 (54.9–59.0)	51.5 (50.8–52.3)	33.3 (31.6–35.1)	22.5 (21.0–24.1)
Sex									
Male	11.8 (11.3–12.3)	6.4 (5.5–7.5)	2.7 (2.2–3.3)	24.7 (24.1–25.4)	53.2 (49.9–56.5)	57.8 (56.0–59.6)	46.8 (46.1–47.6)	21.6 (19.2–24.2)	11.7 (10.4–13.0)
Female	8.6 (8.2–9.0)	4.5 (2.9–6.8)	2.6 (2.0–3.4)	26.1 (25.4–26.7)	53.5 (51.8–55.1)	57.8 (56.1–59.5)	44.9 (44.2–45.6)	20.8 (19.1–22.6)	14.4 (13.1–15.8)
Ethnicity									
Han	10.3 (10.0–10.7)	5.4 (4.6–6.2)	2.7 (2.3–3.1)	26.5 (26.0–27.0)	54.7 (52.5–56.9)	58.4 (56.8–60.0)	46.2 (45.6–46.7)	20.7 (19.6–21.9)	13.0 (12.0–14.0)
Mongolian	4.9 (3.0–7.6)	0.9 (0.2–3.6)	0.4 (0.1–1.7)	11.2 (8.2–14.8)	66.6 (57.1–76.2)	57.5 (52.8–62.2)	23.4 (19.2–27.9)	7.0 (3.6–13.4)	5.7 (2.4–12.7)
Tibetan	11.8 (9.6–14.4)	3.2 (2.4–4.2)	2.6 (1.4–5.1)	8.1 (6.3–10.3)	26.3 (22.9–29.8)	51.2 (42.9–59.5)	53.6 (50.0–57.2)	19.4 (15.3–24.2)	18.2 (13.9–23.4)
Uyghur	2.71 (1.4–4.8)	10.1 (7.6–13.2)	6.0 (1.7–19.2)	11.6 (8.6–15.1)	35.5 (32.4–38.7)	42.6 (25.9–59.2)	22.4 (18.5–26.8)	39.4 (33.5–45.3)	18.2 (14.8–22.1)
Zhuang	13.0 (10.1–16.3)	15.7 (8.3–27.5)	2.0 (0.9–4.4)	28.4 (24.4–32.6)	46.3 (35.0–57.7)	52.8 (39.0–66.7)	56.8 (52.3–61.2)	44.6 (30.3–58.8)	11.0 (6.5–17.9)
Hui	7.5 (5.3–10.3)	2.6 (1.1–5.8)	1.5 (0.5–3.9)	21.9 (18.1–26.0)	47.3 (40.5–54.1)	57.8 (49.7–65.9)	44.8 (40.2–49.5)	12.9 (7.1–22.6)	9.1 (6.3–13.0)
Other	8.6 (7.2–9.7)	6.3 (4.2–9.3)	2.2 (1.3–3.8)	20.1 (19.5–23.5)	42.5 (39.6–45.5)	52.5 (48.3–56.8)	44.0 (40.6–48.7)	23.6 (19.2–28.7)	14.2 (10.3–19.4)
Location type									
Urban	7.9 (7.3–8.5)	4.7 (3.9–5.7)	1.8 (1.5–2.1)	27.1 (26.2–28.0)	57.7 (55.3–60.2)	60.9 (58.5–63.3)	42.9 (41.9–44.0)	20.2 (18.3–22.3)	12.7 (11.4–14.0)
Rural	10.9 (10.5–11.3)	5.7 (4.8–6.7)	3.3 (2.7–4.0)	24.8 (24.3–25.4)	52.0 (49.5–54.4)	55.6 (53.6–57.5)	46.9 (46.3–47.5)	21.6 (20.2–23.0)	13.3 (12.0–14.7)
Region									
Eastern	11.1 (10.5–11.7)	4.9 (4.0–5.9)	2.4 (2.0–2.9)	29.3 (28.4–30.1)	53.7 (51.2–56.2)	59.1 (56.5–61.8)	47.4 (46.4–48.4)	19.1 (17.1–21.3)	12.2 (10.6–14.1)
Central	10.2 (9.7–10.7)	4.6 (3. 8–5.6)	2.2 (1.5–3.1)	26.5 (25.8–27.2)	54.2 (50.4–57.9)	58.8 (56.1–61.5)	46.9 (46.1–47.7)	22.6 (20.7–24.7)	12.1 (10.6–13.8)
Western	9.0 (8.5–9.6)	6.7 (5.3–8.5)	3.4 (2.8–4.3)	19.7 (18.9–20.5)	52.2 (48.4–56.1)	55.1 (53.2–57.1)	42.6 (41.6–43.6)	21.9 (20.2–23.6)	15.0 (13.5–16.6)
Total	10.1(9.8–10.5)	5.5 (4.8–6.3)	2.6 (2.3–3.1)	25.4 (25.0–25.9)	53.3 (51.3–55.3)	57.8 (56.3–59.3)	45.8 (45.3–46.4)	21.2 (20.1–22.4)	13.0 (12.1–14.0)

*HBsAg, hepatitis B virus surface antigen; anti-HBs, antibody to hepatitis B virus surface antigen; anti-HBc, antibody to hepatitis B virus core antigen.
